# Meso-Scale Simulation of Concrete Uniaxial Behavior Based on Numerical Modeling of CT Images

**DOI:** 10.3390/ma12203403

**Published:** 2019-10-17

**Authors:** Haokai Sun, Yang Gao, Xinyu Zheng, Yibo Chen, Zhen Jiang, Zeyao Zhang

**Affiliations:** 1 State Key Laboratory of Geomechanics and Geotechnical Engineering Institute Rock Soil Mechanics, Chinese Academy of Sciences, Wuhan 430071, China; 13903188557@163.com; 2 Hebei Province Key Lab of Structural Health Monitoring and Control, Shijiazhuang Tiedao University, Shijiazhuang 050043, China; jiang654470570@163.com (Z.J.); zhangzeyao_sjz@163.com (Z.Z.); 3 Geotechnical and Structural Engineering Research Center, Shandong University, Jinan 250061, Shandong, China; zhengxinyu_sjz@163.com; 4 Key Laboratory of Transportation Tunnel Engineering of Ministry of Education, Southwest Jiaotong University, Chengdu 610031, China; chenyibojc@sina.com

**Keywords:** aggregate boundary, CT, grain based model

## Abstract

It is important to study the failure mechanism of concrete by observing the crack expansion and capturing key structures at the mesoscale. This manuscript proposed a method for efficiently identifying aggregate boundary information by X-ray computed tomography technology (CT) and a discrete element modeling method (DEM) for equivalent random polygon aggregates. This method overcomes the shortcomings of the Grain Based Model (GBM) which is impossible to establish a mesoscopic model with a large difference in grain radius. Through the above two methods, the CT slice images were processed in batches, and the numbers of edges, axial length, elongation of the aggregate were identified. The feasibility of the method was verified by the comparison between experimental and simulating results. Three mesoscopic models for different porosities were established. Based on aggregate statistics, this manuscript achieved the meso-model recovery to the maximum extent. The test results show that the crack appeared at the tip of the aggregate firstly, and then the broken boundary was applied in the direction of the applied load and around the pores. Finally, the crack was selectively expanded under the axial force. During the loading process, the minor principal stress was normally distributed. As the porosity and loading time increased, the heterogeneity increased.

## 1. Introduction

The grain-scale microstructure controlled the micromechanical behavior and governed the complex macroscopic response [1]. It is well-known that the heterogeneity (mortar, aggregate and the interfacial transition zone) plays a key role in the generation of localized tensile stress, which further results in crack initiation. At the mesoscopic level, i.e., between the microscopic scale and the macroscopic scale (10^−6^ m–10^−0^ m), the interfacial transition zone (ITZ) between mortar and aggregate is the weak part of concrete, which determines the concrete strength. However, the failure mechanism was still unclear. Since concrete is multi-composite material, the stress state of the mesostructure is significantly different from that of the macro-structure. Therefore, it is necessary to study the macroscopic failure mechanism and characteristics of strength and deformation of concrete at the mesoscale. Technologies such as the X-ray computed tomography technology (CT) and scanning electron microscopy are used to reconstruct mesoscale models and perform numerical simulations. Through these technologies, microcracks and even macroscopic damage are easily monitored. With the increasing application of concrete, it becomes more important to study its mechanical properties by numerical simulation. The performances of mesoscale concrete were investigated by the early-age shrinkage cracking process of high-performance concrete [2], impact performances of reinforced concrete beams [3], spatial variability of chloride and its influence on the thickness of concrete cover [4], the effective thermal conductivity of tensile cracked concrete [5,6], and the behavior of concrete under high confinement [7], respectively.

In general, the finite element method (FEM) is difficult to simulate the crack initiation and expansion behavior of materials. However, on the basis of this method, the extended FEM was developed to simulate the extended expansion behavior of cracks. Moreover, the nonlinear displacement mode was introduced to describe crack displacement [8]. This method is difficult to operate, and the obtained results show poor agreement with the actual results. In addition, the discrete element method (DEM) is used to describe the motion state and interaction of a non-continuous body. Especially, only the block and contact parameters are involved in the simulation process. Two typical types of software are the particle flow analysis program and the block discrete element general program. In the numerical simulation study, the first step was to calibrate the microscopic properties of conventional rock [9]. However, there are some existing limitations. When the particle flow code (PFC) was employed to calibrate the unconfined compressive strength, it significantly overestimated the tensile strength of the rock material with an approximate 0.25 ratio of predicted Brazilian tensile strength (BTS) to unconfined compressive strength (UCS) [10]. Moreover, an intrinsic problem of the PFC approach was that a minimum porosity of 9% had to be incorporated when simulating the low-porosity rock (<2%) due to the circular shape of the particles [11]. The methods for simulating the mesostructure include PFC, square particles of Realistic Failure Process Analysis (RFPA2D), finite element meshes [4], models for elliptical and polygonal aggregates, disk-shaped particles [12,13], triangular particles of different element codes (block discrete element) [14], and the stochastic distribution of properties (e.g., Weibull distribution) [15]. In view of geometry, the polygonal structure was representative of the mineral structures observed in the crystalline rock. The conventional polygonal structure was usually generated in block discrete element using the Voronoi tessellation technology [11].

Although several established three-dimensional (3D) models are available [16,17], this study is still conducted using a 2D model, according to the following considerations: (1) 2D modeling results were in good correspondence with the results obtained from the 3D simulation of spall tests [18]; (2) as a large number of samples were required for statistical analysis, a long calculation time using the 3D model was unacceptable. [4]; (3) a few papers [19,20] indicated that the qualitative analysis could be achieved by 2D models, such as identifying the most important influence parameters or providing valuable information for further 3D analysis. From a morphological point of view, it is unscientific to characterize the 3D aggregate distribution using only the 2D aggregate distribution. However, the expansion form of the concrete crack is affected by the randomness of the aggregate size inside the concrete, the spatial distribution of the aggregate, the long axis direction, and the form of the void distribution, respectively. Thus, it is difficult to comprehensively analyze the causes of crack propagation according to 3D CT image modeling, and it also increases the computer computing burden. Therefore, this paper has aimed to generate a 2D model regarding the probability and statistics method, and to analyze the micro-crack propagation law at the mesoscale.

This manuscript proposed a method for efficiently identifying aggregate boundary information in CT images and DEM method for equivalent random polygon aggregates. This method overcomes the shortcomings of GBM which is impossible to establish a mesoscopic model with a large difference in grain radius. Through the above two methods, the CT slice images were processed in batches, and the numbers of edges, equivalent radius, angle and Elongation ratio of the aggregate were identified. The feasibility of the method was verified by the comparison between experimental and simulating results. Three mesoscopic models for different porosities were established. The change process of cracks in concrete was studied by observing the change of the model under uniaxial stress.

## 2. CT Image Process and Selection and Optimization of Aggregate Boundary Coordinates

In 1967, Hounsfield developed the first CT scanner to identify the exact location of the internal structure of a non-transparent object. Since then, the CT technology has been widely applied in the bioengineering, geological engineering, geotechnical engineering and materials engineering. Image processing technology was used to analyze the characteristics of aggregates statistically by processing the 2D slice images of concrete, such as the edge number, area, equivalent radius, elongation ratio, and size distribution. Based on aggregate statistics, this manuscript achieved the meso-model recovery to the maximum extent from 3D to 2D. However, since the boundary information collected for the first time has more noise, this makes the numerical model require too many units to describe. But this is not conducive to the operation of the computer. It is essential to simplify the collected boundary information and achieve the same mechanical properties.

### 2.1. Experimental Procedure

**Figure 1:**
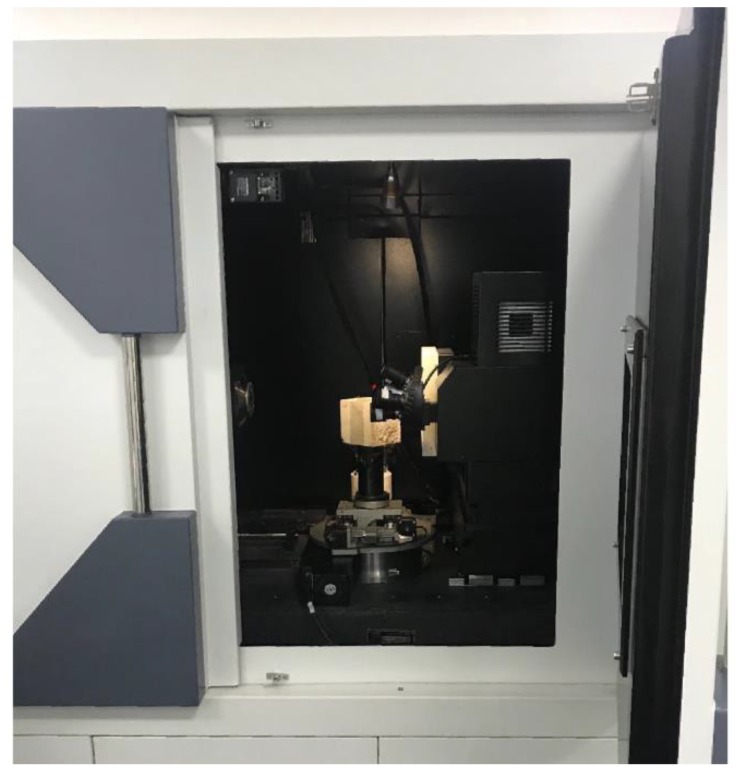
CT equipment.

In this test, batching was carried out by weighing a precalculated amount of concrete constituent according to the mix ratio of 1 : 2.58 : 3.82 : 0.62 : 0.3 (cement : sand : coarse aggregate : water : fly ash). Portland cement of type 42.5 N and Class F fly ash were used as cementitious materials. The mixture was poured vertically into a mold of 100 mm × 100 mm × 100 mm. Use a vibrating table to fully vibrate to minimize the effect of initial bubbles in the concrete on the test piece. In the production process, in order to minimize the influence of the initial bubbles, the concrete was appropriately shaken on the vibration table. The CT tests were carried out at the College of mining and safety engineering, Shandong University of Science and Technology, as shown in Figure 1. A total of 1200 slices were obtained for each scan, which included 1599 × 1599 × 1200 pixels. The size of each pixel was 0.08732747 mm × 0.08732747 mm.

### 2.2. Aggregate Collection Selection and Boundary Coordinate Determination

Concrete is composed of aggregate and mortar. Since the layout of the mortar is completely dependent on the distribution of aggregates, it is unnecessary to consider the mortar distribution alone. Different materials, such as aggregates, mortars, and voids, the pixel points formed in the CT image, have different attenuation coefficients or absorption coefficients of CT. Thus, the corresponding numbers from 0 to 255 in the digital matrix accord with a small square of unequal gray scales from black to white.

**Figure 2:**
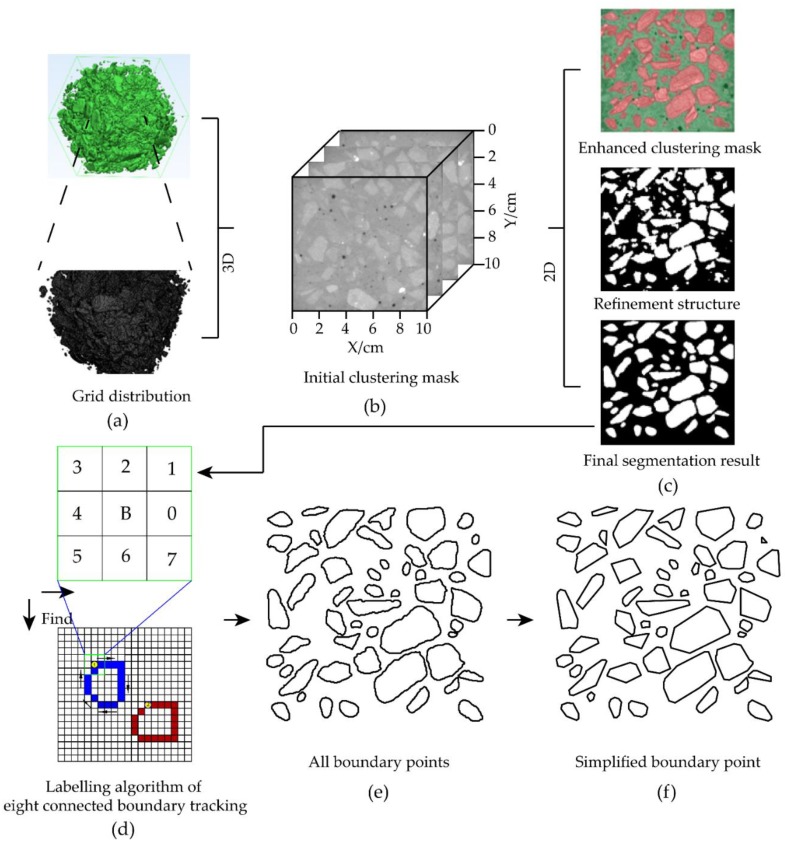
Extraction process of aggregates in CT images: (**a**) 3D modeling; (**b**) CT slice in the initial state; (**c**) Process of selecting aggregate; (**d**) Eight connected boundary tracking; (**e**) All boundary points; (**f**) Simplified boundary point.

Pre-processes of smoothing and denoising were firstly carried out on the CT image, as shown in Figure 2. The extraction of the aggregate and mortar area was then performed by the Trainable Weka Segmentation function module of the Image J from the open source image analysis tool (The default is mortar including voids). When an area in the recognition image satisfies the predetermined requirement, the area can be segmented. If it is not well recognized, the clustering conditions need to be further increased. Figure 2c describes the selected dark red region is aggregates, and the green region is the mortar. At the edge of the segmented image, some aggregates and mortar were not successfully segmented, which required a further refinement of the clustering criteria [21]. The clustering criteria can be applied to all sliced images in the testing block of concrete. It can be seen from Figure 2 that there are still noises, boundary connection domains and unselected aggregates, which seriously affect the later statistics. Therefore, smoothing, etching, and expansion operations, as well as denoising process, were carried out on the edge of the aggregates. Figure 2 presents the final extraction results of aggregates.

At the same time, the CT image was subjected to the 3D reverse reconstruction, which experienced the processes of smoothing-reducing-auto remeshing-quality preserving reduce triangles. The obvious advantage of this method is that the internal structure of the testing block can be restored as a proportion of 1 : 1 in Figure 2. However, due to the limitations of CT equipment and image processing technology, it is deduced that the number of connected aggregates is too large, and the surface roughness is great, which results in a huge number of grids to describe the shape. Since the number of units in the 0.001 m^3^ testing block is more than 10,000 in Figure 2, it is difficult to conduct a statistical study on the concrete damage analysis.

### 2.3. Determination of Boundary Coordinates

After the recognition system completes a batch of CT image processes, a binary image containing the foreground as 1 and the background as 0 is obtained. In this study, the aggregate was 1, and the mortar was 0.

When P (x, y) is a boundary point of the object, the next boundary point of P (x, y) must be within its eight neighboring locations. Thus, the boundary tracking can be achieved according to the eight neighborhood information. First, the object area was searched both from top to bottom and left to right until the boundary point as the starting point was found. Then, the eight neighborhood locations of this point were searched until the next boundary point was found, and the coordinates of the searched pixels were recorded. After that, the searching process was repeated until back to the starting point. Finally, after finishing the search for the closed area, the obtained complete boundary coordinates were arranged clockwise or counterclockwise, as shown in Figure 2.

The closed boundaries produced were special and skipped when searching for the next boundary. The coordinate information of the remaining boundaries was obtained by the above method as well. Finally, we acquired the coordinate information of all closed boundaries in the regular arrangement, as shown in Figure 2. Therefore, an important step was completed in the modeling of digital images.

### 2.4. Optimisation of Image Recognition

**Figure 3:**
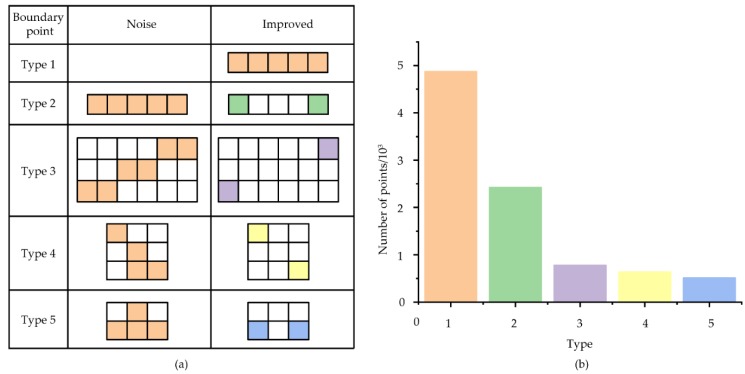
The type and number of noise removed from the boundary: (**a**) Optimized type; (**b**) Number of optimized boundary points.

Although the boundaries of eight neighborhoods can be chosen in a particular order, the selected number of boundary points is quite large. Moreover, as the pixel itself occupies an integer coordinate point, it is extremely easy to generate a jagged boundary. Therefore, the noise reduction and denoising algorithm were written by Matlab according to the tracked boundaries of eight neighborhoods. The types of identified noises included: other points on the same line except the endpoint (Type 1), a midpoint of an adjacent boundary with a similar slope (angle difference less than or equal to 5°) (Type 2), a jagged boundary point (Type 3), one of the edges of a side with a pixel length of less than 3 (Type 4), and a sharp point (Type 5). The type and number of noise was shown in Figure 3.

### 2.5. Probability Statistics of Geometric Features of Aggregates

At present, many modeling methods only require matching the area or consider the number of lengths, the elongation, and the equivalent radius of aggregates. However, the internal relations of these parameters are obviously ignored. For example, the deformation and failure mechanism of the aggregate with a long equivalent radius is intrinsically different from those of two or more aggregates with short equivalent radii of concrete. The essence of the mesoscopic model is to simulate the real situation to the maximum extent. Therefore, the identification process is further refined based on previous modeling methods.

The aggregate area fraction of the total concrete area was considered as roughly 30%. Furthermore, to reconstruct the mesoscopic model accurately, the aggregates were divided into three gradations, according to the lengths of all the equivalent radius in 1200 CT images: the first grading is 0–1 cm, the second grading is 1–2 cm, and the third grading is 2–3 cm. The probability distributions of the aggregate quantity, edge number, elongation ratio, angle and equivalent radius in each gradation were analyzed, respectively. These parameters control the number of sides, length/width ratio, long axis direction and average length of the aggregate. In this way, the relationship between the equivalent radius and other parameters was established. The statistical results are shown in Table 1, Table 2 and Table 3. The related research mentioned that the number of aggregates in the CT image of C30 concrete followed a Gaussian distribution, and each aggregate area, equivalent radius, and elongation followed a lognormal distribution [22].

Since each aggregate coordinate information is separately placed in a matrix during the boundary reading process, equivalent radius, edge number, angle, elongation ratio and center coordinate of each aggregate can be quickly calculated during the information statistics (Center coordinates are in random distribution). Besides, the equivalent radius is divided into 3 or more groups from large to small. The probability of the aggregate information in each group is calculated, and then the probability density functions are also obtained. Therefore, the model is successfully established to represent the true aggregate distribution according to the steps in the placement process.

## 3. Aggregate Formation and Placement Process

### 3.1. Optimisation of the Generation and Placement Process

In this section, a polygon random aggregate model was generated. The geometry generation process was improved based on the algorithm described by Wang [23]. The length of the aggregate was divided into several groups from large to small. The probability density functions were constructed, such as the long axis direction, the number of edge and elongation rate. The specific methods for improving the production of aggregates are as follows:

(1) $$\varphi_{i}=\frac{2\pi }{n}+\left({2\eta }_{i}-1\right)\times \delta \times \frac{2\pi }{n}$$

where $\eta_{i}$ is a random number distributed between 0 and 1 uniformly, δ is an arbitrary value less than 1. To ensure the closed polygon, the sum of all subtended angles should equal to 2π. The subtended angles are modified as

(2) $$\bar{\varphi_{i}}{=\varphi }_{i}\times \frac{2\pi }{\sum_{j=1}^{n}\varphi_{i}}$$

Then the polar angles $\theta_{i}$ can be calculated by

(3) $$\theta_{i}=\alpha +\overset{i-1}{\underset{j=1}{\sum }}\bar{\varphi_{i}}$$

where α is a phase angle determining the orientation of the particle.

Then the polar axis length $r_{i}$ can be calculated by:

(4) $$r_{i}{=A}_{0}+\left({2\eta }_{i}-1\right){\times A}_{1}$$

in which $A_{0}$ is the average radius, $A_{1}$ is the extreme value in the variation of the average radius,

The coordinates of each aggregate vertex:

(5) $$x_{i}{=x}_{0i}{+r}_{i}{\cdot cos\theta }_{i}$$

(6) $$y_{i}{=y}_{0i}{+r}_{i}{\cdot sin\theta }_{i}$$

in which $x_{0i}$ is the x-coordinate of the random point coordinates within the specified range, $y_{0i}$ is the y-coordinate.

The angle of rotation required by the angle between the longest axis of the aggregate and the horizontal direction is recorded as *µ*. To control the angle of the long axis of the aggregate equaling the obtained angle, the shape of the particle is adjusted by the matrix change in the Cartesian coordinate system:

(7) $$X^{\prime }={\left[\begin{array}{ccc} 1 & 0 & {-x}_{0i}\\ 0 & 1 & {-y}_{0i}\\ 0 & 0 & 1 \end{array}\right]}^{-1}\left[\begin{array}{ccc} cos\mu & sin\mu & 0\\ -sin\mu & cos\mu & 0\\ 0 & 0 & 1 \end{array}\right]\left[\begin{array}{ccc} 1 & 0 & -1\\ 0 & 1 & -1\\ 0 & 0 & 1 \end{array}\right]X$$

To control the length/width ratio equaling to the obtained elongation ratio (*R*), the shape of the particle is adjusted by the matrix change in the Cartesian coordinate system.

(8) $$X^{\prime \prime }={\left[\begin{array}{ccc} 1 & 0 & {-x}_{0i}\\ 0 & 1 & {-y}_{0i}\\ 0 & 0 & 1 \end{array}\right]}^{-1}\kappa \left[\begin{array}{ccc} cos\mu & sin\mu & 0\\ -sin\mu & cos\mu & 0\\ 0 & 0 & 1 \end{array}\right]\left[\begin{array}{ccc} 1 & 0 & -1\\ 0 & 1 & -1\\ 0 & 0 & 1 \end{array}\right]X$$

in which κ is a probability function about elongation ratio.

If the grading curve is given by P(*D*) in which *D* is the aperture size of the sieve and P(*D*) is the cumulative percentage passing the sieve. The area of aggregate within the grading segment [*D_s_, D_s+1_*] is calculated as:

(9) $$A_{agg}\left[D_{s}{,D}_{s+1}\right]=\frac{P\left(D_{s+1}\right){-P(D}_{s})}{P\left(D_{max}\right){-P(D}_{min})}{\times R}_{agg}{\times A}_{con}$$

where $D_{min}$ is the minimum size of aggregate, $D_{max}$ is the maximum size of aggregate, $A_{con}$ is the area of concrete and $R_{agg}$ is the area ratio of coarse aggregate. Aggregate placement process starts with the grading segment containing the largest size particles.

When placing the aggregates into the concrete section, two standards must be met: firstly, it must be within the boundaries of the concrete zone completely; secondly, it must not overlap with the previously placed aggregate [22,23]. In the process of placing aggregates, previous studies have reported that the circumscribed circle was instead of the aggregate boundary to determine the intersection and the replacement of the aggregate area. As a result, the compact aggregate could not be produced, and the calculation error was large. Matlab using the custom function was used to build an accurate model using real aggregate boundaries and areas.

### 3.2. Voronoi Generation Process

Voronoi has been widely used to divide and calculate microstructures [24,25,26]. Rock failure was captured in terms of plastic yielding of the rock matrix, displacements of the discontinuities using the polygonal block, and Voronoi tessellation, respectively. This study employed the Voronoi division to divide the discrete element mesoscopic model. In the Voronoi division process, a set of discrete points (*X_i_*, *Y_i_*) was set (i=1, 2, 3, ···, k, k was the number of discrete points) in the plane area. Adjacent polygons of number K in this area were divided by following the below requirements:

(1) Each polygon contains only one discrete point;
(2) If any point (*X’, Y’*) is in a polygon with discrete points (*X_i_, Y_i_*). Inequality 10 should always be satisfied when *i≠j*;
  (10) $$\sqrt{{{(X}^{\prime }{-X}_{i})}^{2}{+(Y}^{\prime }{-Y}_{i}{)}^{2}}<\sqrt{{{(X}^{\prime }{-X}_{j})}^{2}{+(Y}^{\prime }{-Y}_{j}{)}^{2}}$$
(3) If the point (*X’*, *Y’*) is on the common side of two polygons with discrete points (*Xi*, *Yj*) and discrete points. Then the equation 11 should always be satisfied;
  (11) $$\sqrt{{{(X}^{\prime }{-X}_{i})}^{2}{+(Y}^{\prime }{-Y}_{i}{)}^{2}}=\sqrt{{{(X}^{\prime }{-X}_{j})}^{2}{+(Y}^{\prime }{-Y}_{j}{)}^{2}}$$

The resulting polygon is called Voronoi. A triangle formed by connecting discrete points in every two adjacent polygons with a straight line is called a Voronoi triangle.

### 3.3. Improvements to the GBM Method

The GBM uses Particle Flow Code (PFC) to generate a series of circular particles. A uniformly distributed particle model was built according to the desired mortar unit sizes. After sufficient operations, the original spheres were evenly distributed throughout the area, and there were contacts between adjacent spheres. The spherical coordinate information in the mortar and aggregate regions obtained by the CT image processing system was recorded separately. Then, a polygonal network was generated by joining the centroids with lines. The generated grain structure, described by a 2D mesh consisting of nodes, edges and elements, was then exported from PFC and imported into block discrete element model.

**Figure 4:**
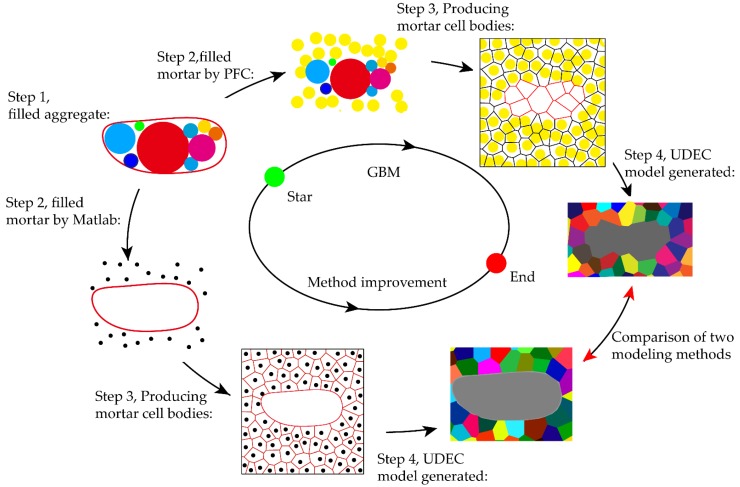
GBM generation and improved method.

GBM was greatly limited due to the complex operation and narrow scopes of application. This method has difficulty modeling the mortar and aggregate at the mesoscale and under various complex situations, such as the difficulties of achieving a dense distribution of polymers along different paths and a smooth boundary with large grain difference radius, implementing complex function operations, and constructing a model based on a statistical probability function. This method is not suitable for establishing a model with a large difference in grain size, and a jagged boundary appears at the larger boundary of the grain, as shown in Figure 4. In order to meet the requirements of modeling, GBM is improved according to this difference. This improvement is not limited to the use of PFC to generate particles. This process can be performed using any programming software. When generating a description of the mortar particles, it is not generated by the boundary of the grain describing the aggregate. Instead, particles are generated in the difference between the true aggregate boundary and the concrete boundary, and the void centroids are calculated and Voronoi is generated.

**Figure 5:**
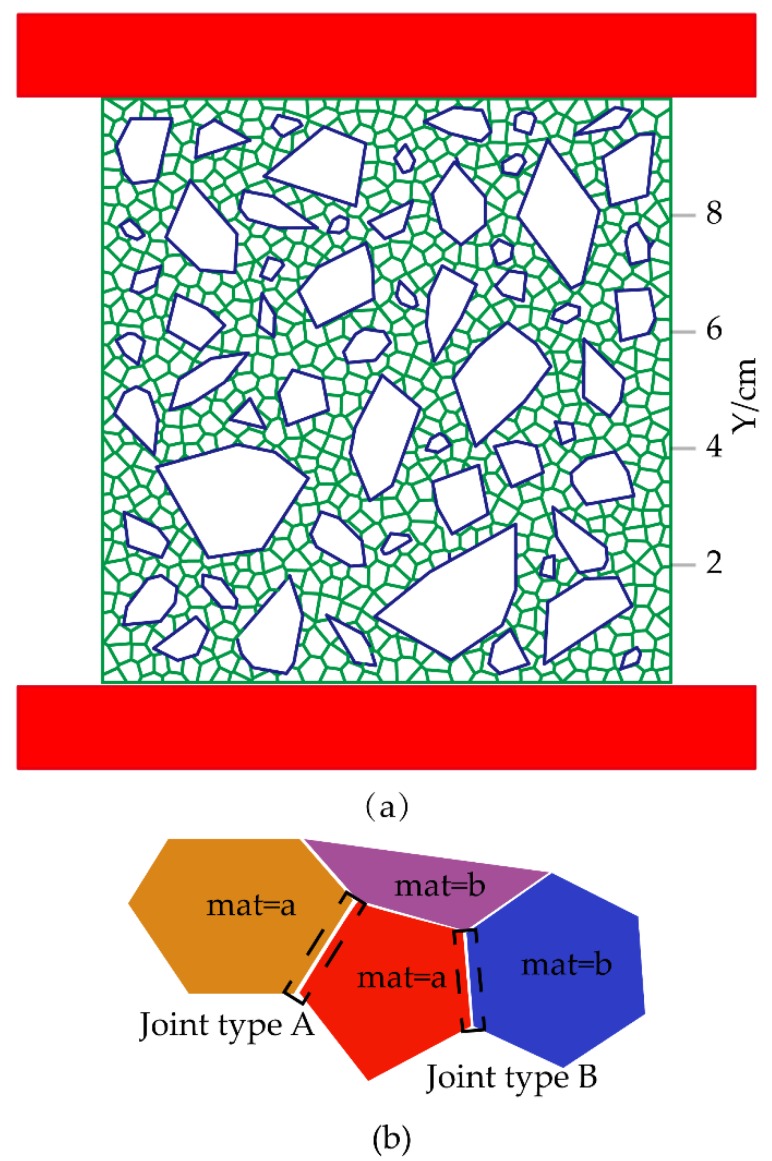
Improved process of mesoscale modeling method: (**a**) Concrete meso-scale models; (**b**) Joint assignment method.

Therefore, the modeling process was improved to solve the problems of mesoscale modeling, as follows:

(1) In the mortar area, discrete points of specific density are randomly generated by Matlab to meet the required engineering conditions. For example, if the analysis object is an isolated mortar, the discrete points from top to bottom become increasingly sparse, indicating that the upper portion is prone to cracking.
(2) If the internal crack of the aggregate is considered, discrete points are also generated inside the aggregate. The numerical model is shown in Figure 5a, which shows a uniform distribution.

### 3.4. Model Parameter Assignment Method

The properties of mortar materials were assigned to the entire model, and aggregate parameters were assigned to the block at the coordinate center of aggregates. The material properties of joints were defined by determining whether the material parameters of the left and right blocks were consistent. If consistent, the joints were prefabricated with the mortar parameters (a = b); otherwise, the parameters of the interface between the aggregate and the mortar were set for joints (a ≠ b), as shown in Figure 5b. When assigning values to all contacts, the first step was to determine whether the material properties of the blocks on both sides of the joints were consistent during the traversal of all joints. If consistent, the joint property was the inter-mortar contact property; otherwise, the property was the aggregate and mortar contact property.

## 4. Test Verification and Study on Mechanism of Crack Propagation

### 4.1. Verification of Uniaxial Compression Test on Concrete

**Figure 6:**
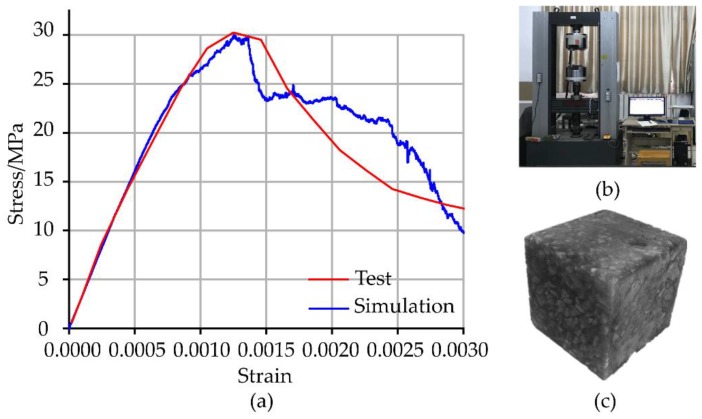
Uniaxial test.

The above statistical results of C30 concrete by the CT image processing were firstly selected to verify the correctness of the CT image processing, aggregate generation and model construction process. Meanwhile, the numerical model was established using the improved modeling method. The model size was 100 mm × 100 mm, as shown in Figure 6. The material properties in the model are shown in Table 4. In addition, a sensitivity analysis was conducted to study the effect of loading rate on the modeling method, which indicates that the loading rate is sufficiently small to assure that the model remains in the quasi-static condition. However, it should be observed that in the post-peak branch, the model predicts a degradation of the strength and of the stiffness more severe than in the experimental test [27].

Figure 6 shows the stress-strain behavior in the uniaxial compression test. It can be seen that when the strain reaches 0.14%, the concrete reaches the peak stress of 30 MPa. The testing results before the peak stress show good agreement with the simulation results. However, the stress-strain gap is larger after the peak, which is mainly caused by the following reasons:

There are tiny pores in the concrete; the strength distribution of the contact surface of the mortar and aggregate is not uniform; the influence of the selected mechanical model.

### 4.2. Analysis of the Micro-Fracture Expansion Mechanism of Concrete

In the simulation process, the acoustic emission (AE) function was designed by measuring the number, type, angle and position of cracks to reveal the laws of crack initiation and expansion in concrete. Since a consistent trend was found in the characteristics of the acoustic emission and energy, they were studied together. The real-time spatial positioning of different types of cracks was also available [30].

With the obtained Young’s modulus, the bulk modulus *K* and shear modulus *G* for the blocks in the numerical model can be calculated with the following equation:

(12) $$K=\frac{E}{3(1-\mu )}$$

(13) $$G=\frac{E}{2(1+\mu )}$$

where *E* and *μ* are Young’s modulus and Poisson’s ratio, respectively.

The normal stiffness and shear stiffness for the contacts in the numerical model can be derived from the equations [31]:

(14) $$k_{n}=factor\times max[\frac{K+(4/3)G}{\Delta Z_{min}}]$$

where $\Delta Z_{min}$ is the smallest width of the zone adjoining the contact in the normal direction and factor is a multiplication factor (usually set to 10)

From the normal direction to a contact, the stress-displacement relation is assumed to be linear and governed by the stiffness $k_{n}$ [31]

(15) $$\Delta \sigma_{n}{=-k}_{n}\Delta u_{n}$$

where $\Delta \sigma_{n}$ is the effective normal stress increment and $\Delta u_{n}$ is normal displacement increment.

There is also a tensile strength limit, T, for the joint. If the tensile strength is exceeded (i.e., if $\Delta \sigma_{n}$ < −T), then $\sigma_{n}=0$. Similarly, in shear, the response is controlled by a constant shear stiffness, $k_{s}$ The shear stress, $\tau_{s}$ is limited by a combination of cohesive (*c*) and frictional (*φ*) strength [31].

Thus, if

(16) $$\left|\tau_{s}\right|\leq {c+\sigma }_{n}{tan\varphi =\tau }_{max}$$

then

(17) $$\Delta \tau_{s}{=-k}_{s}\Delta u_{s}^{e}$$

Or if

(18) $$\left|\tau_{s}\right|\geq \tau_{max}$$

Then

(19) $$\tau_{s}=sign(\Delta u_{s}^{e}{)\tau }_{max}$$

where $\Delta u_{s}^{e}$ is the elastic component of the incremental shear displacement; and

$\Delta u_{s}$ is the total incremental shear displacement.

The parameter calibration method is the same as GBM, and the uniaxial compression test of the pure mortar test block and the Brazilian splitting test were used to calibrate the microscopic parameters. The final determined parameters are shown in Table 4. The macroscopic crack propagation state obtained by the simulation are the same as the experimental results, which proves the rationality of the microscopic parameters.

The macroscopic fracture of the concrete is the result of accumulation of damage and breakage of the microcell. The damage variable of concrete is defined based on the theory of damage mechanics:

(20) $$D=\frac{S_{D}}{S_{O}}\times 100\%$$

where $S_{D}$ and $S_{O}$ are the number of joint nodes in damage and all joint nodes, respectively.

According to the damage variable, the destruction process was classified into three stages. The first stage was the compaction stage, which was characterized only by compression without cracks. The second stage and the third stage were respectively a large amount of contact in the ITZ and the mortar. It can be seen that the micro-cracks in ITZ develop rapidly in the early stage and the curve is convex downward. When the contact in the mortar is broken, the contact failure speed in the ITZ is slowed down, and the curve is concave. The damage degree of ITZ is much greater than the damage degree of mortar.

**Figure 7:**
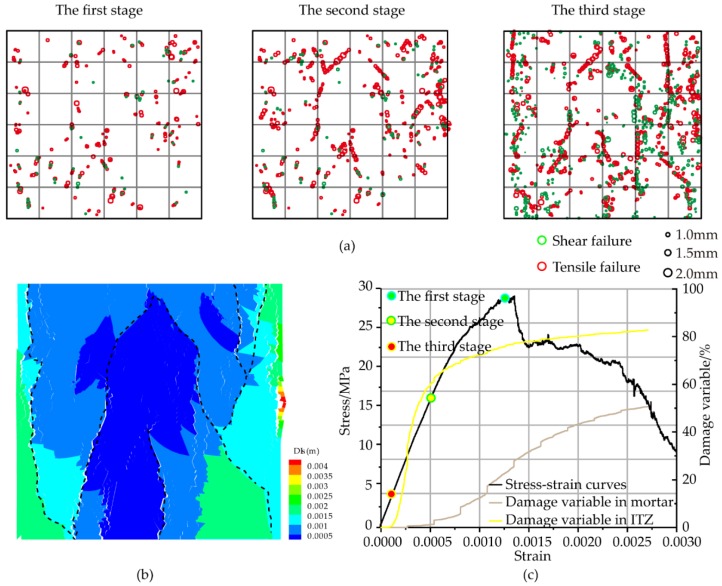
Numerical simulation results: (**a**) Spatial distribution of crack locations (The radius represents the length of the crack, and the color is used to distinguish type of crack.); (**b**) macroscopic fracture distribution in failure state; (**c**) damage and dilation development.

Many scholars have studied the contact surfaces under the meso-structure [32,33]. The damage happened easiest at the tip of the aggregate. Long cracks (length greater than 1 mm) were usually accompanied by micro-cracks (lengths around 0.5 mm). The crack between the mortars began to appear in second stage, but its number was always less than the total number of acoustic emissions in the ITZ. As the loading increased, the length of micro-cracks constantly increased from the original 1.21 mm to 1.47mm. In third stage, the sharp crack expansion appeared, which was characterized by a sharp increase in the number of cracks between mortars. The crack between ITZ developed slowly because the contact base between the mortars was much larger than the aggregate-mortar contact base. The distribution of cracks was linear, which indicated that cracks occurred throughout Stage III; the post-peak stage was characterized by the rapid development of macro-fractures. The final form of macroscopic damage was shown in Figure 7, which was similar to the corresponding study. It can be concluded that the proposed modeling method is feasible. Figure 7b illustrates the initiation and propagation of damage at final stages. Under unconfined compression, the sample failed through a combination of shear failure in the upper right and extension or splitting in the lower part, together with localized splitting in the upper right corner and middle part of the sample. The failure pattern is similar to the experimental results. It can be concluded that the predicted concrete behavior is reasonable and accurate.

### 4.3. Effect of Porosity

**Figure 8:**
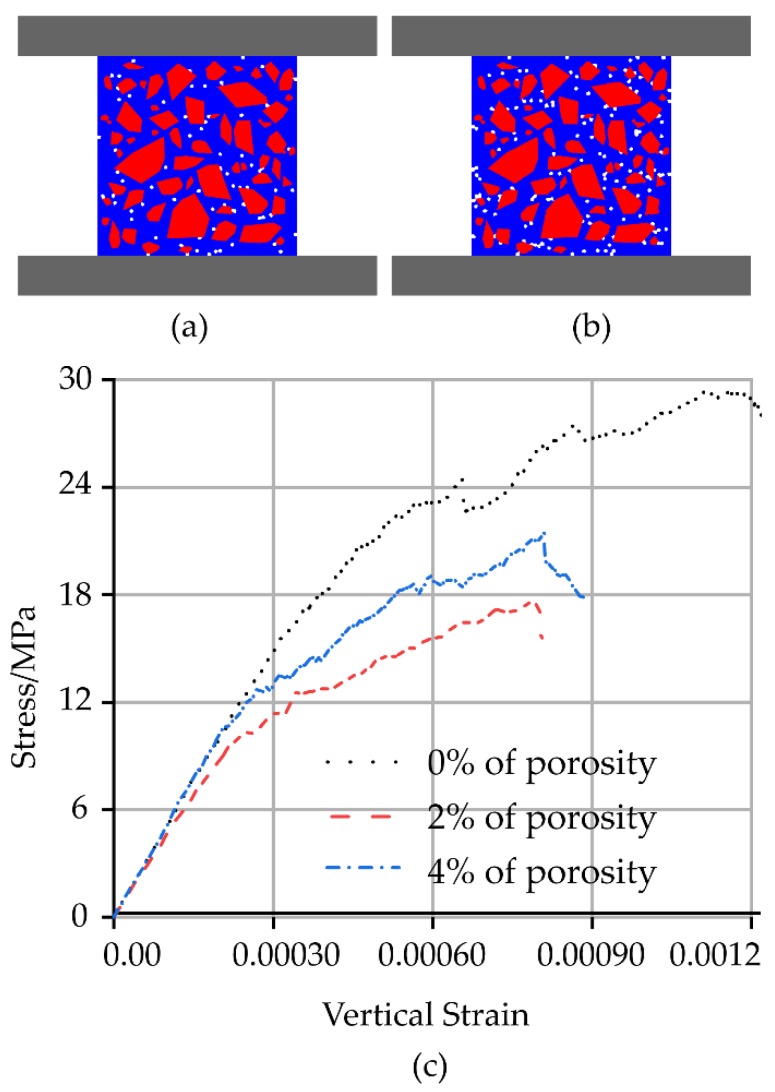
Effect of porosity on the fracture pattern and mechanical behaviour under unconfined compression: (**a**,**b**) Random concrete meso-scale models with a porosity of 2% and 4%; (**c**) Axial stress-strain curves.

Further, the evolution of tension and shear cracks in ITZ and mortar was studied in the concrete with pores. As shown in Figure 8, a fish function with a random number generator that specified the location of the voids was developed and used to construct a sample with a porosity of 2% and 4% by randomly deleting voids until the specified porosity was achieved. These models were used to simulate uniaxial compression experiments, and the results were compared to evaluate the effect of pre-existing pores. It can be seen from the stress-strain diagram that the pre-existing pores have a considerable influence on the performance of the concrete. When the porosities were 2% and 4%, the UCS were 73% and 58.3% of the original compressive strength, respectively. With the increase of porosity, the elastic modulus and strength of the material were significantly reduced, which was consistent with experimental results.

**Figure 9:**
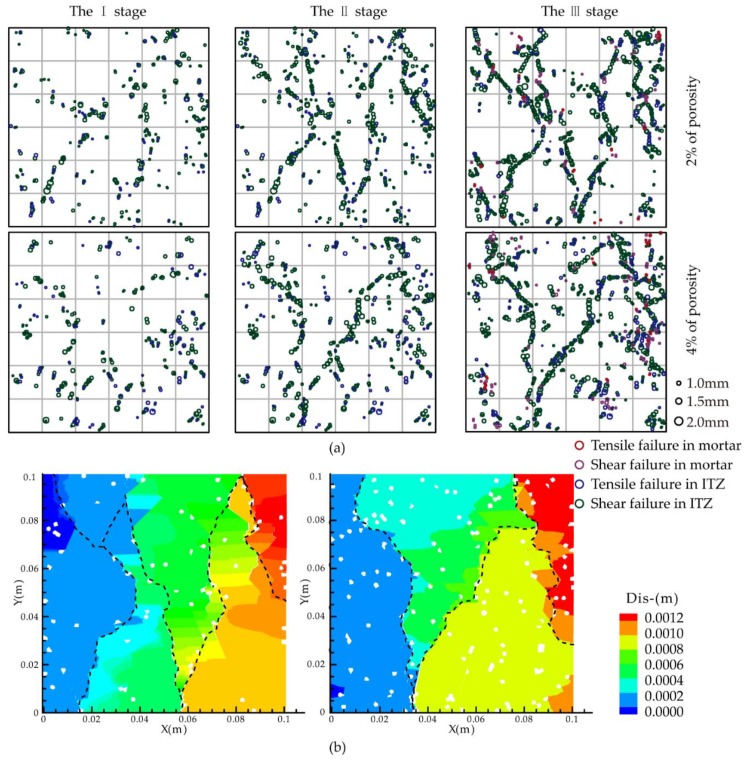
Destruction process at 2% and 4% porosities: (**a**) AE spatial distribution at I-III stages; (**b**) Macroscopic fracture distribution in failure state.

**Figure 10:**
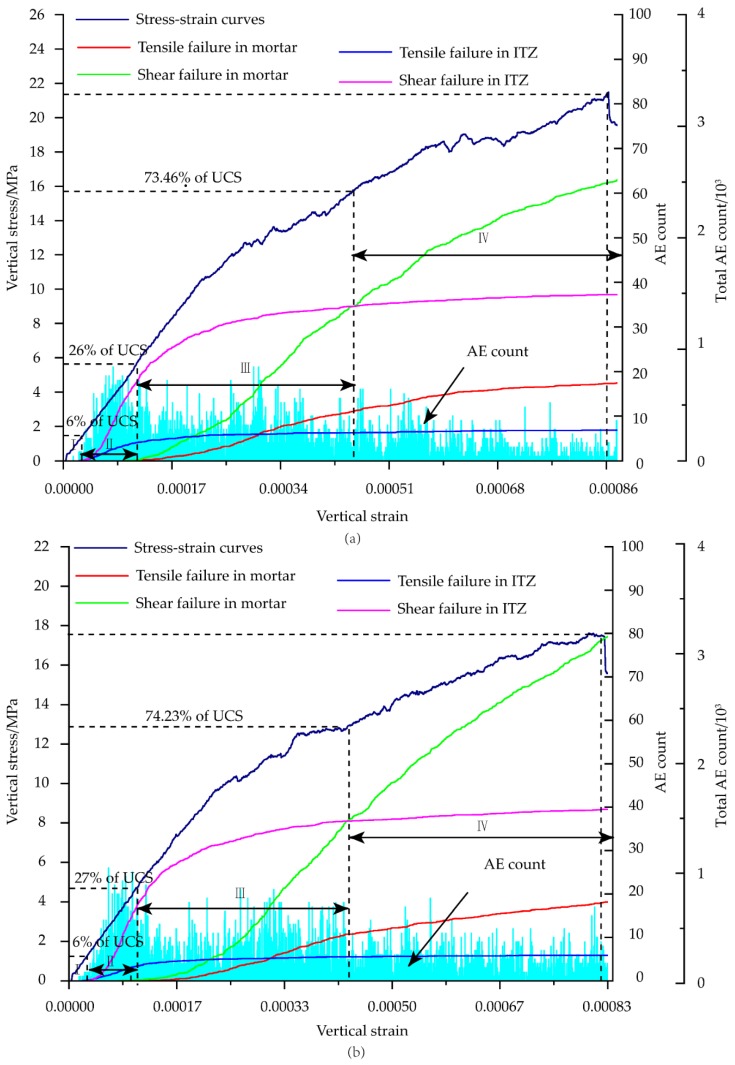
AE development process at (**a**) 2% and (**b**) 4% porosities.

Figure 9 and Figure 10 show the uniaxial compression experiments of concrete with the porosities of 2% and 4%, including stress-strain, the number of acoustic emission rings, tension and shear fracture curves of mortar contact, contact between mortar and aggregate, and shear fracture curve. Similar to the failure process of concrete without porosity, the compression process was divided into four stages. The crack was not produced in Stage I. Stage II was the failure stage of the contact surface between the aggregate and the mortar, in which the only fracture occurred. The shear fracture developed fast, and the number of tensile fractures was about one-third that of shear fractures. The failure threshold was divided into 6.38% and 6.90% of the respective UCS. This indicates that during the failure process, the microcracks first appear in the contacts in the ITZ due to the weak property, and the shear damage is the main type of damage. In Stage III, the contact fracture inside the mortars primarily took place. Microcracks began to appear inside the mortars owing to the continuous application of uniaxial pressure. The contact between aggregate and mortar was not fully destroyed, resulting in a slower growth rate of the damage inside mortars. Shear failure was still the primary failure mode, and the number ratio of shear and tensile failures was approximately 1 : 2. The initial threshold of failure is 26.50% and 26.89% of the UCS. Stage IV was the stage of rapid destruction. The contact in the ITZ was totally broken, and the shear crack inside the mortars increased sharply. The cracks expanded to form macroscopic cracks, causing changes in macroscopic properties (elastic modulus). As a result, the initial thresholds of failure were 73.46% and 74.23% of the UCS.

In Stage II, the crack first appeared at the tip of the aggregate because of the heterogeneity of the mortar and aggregate materials. Then it expanded to the aggregate boundary in the same direction as loading. In Stage III, shear cracks inside mortars occurred at the pores and the tip of the aggregate firstly. The crack near the cavity was extremely easy to penetrate the cracks near the adjacent aggregate and other voids. In Stage IV, the crack generation was not limited around the pores and aggregates as the loading process advanced. Instead, it penetrated the surrounding cracks until the stability was destroyed.

**Figure 11:**
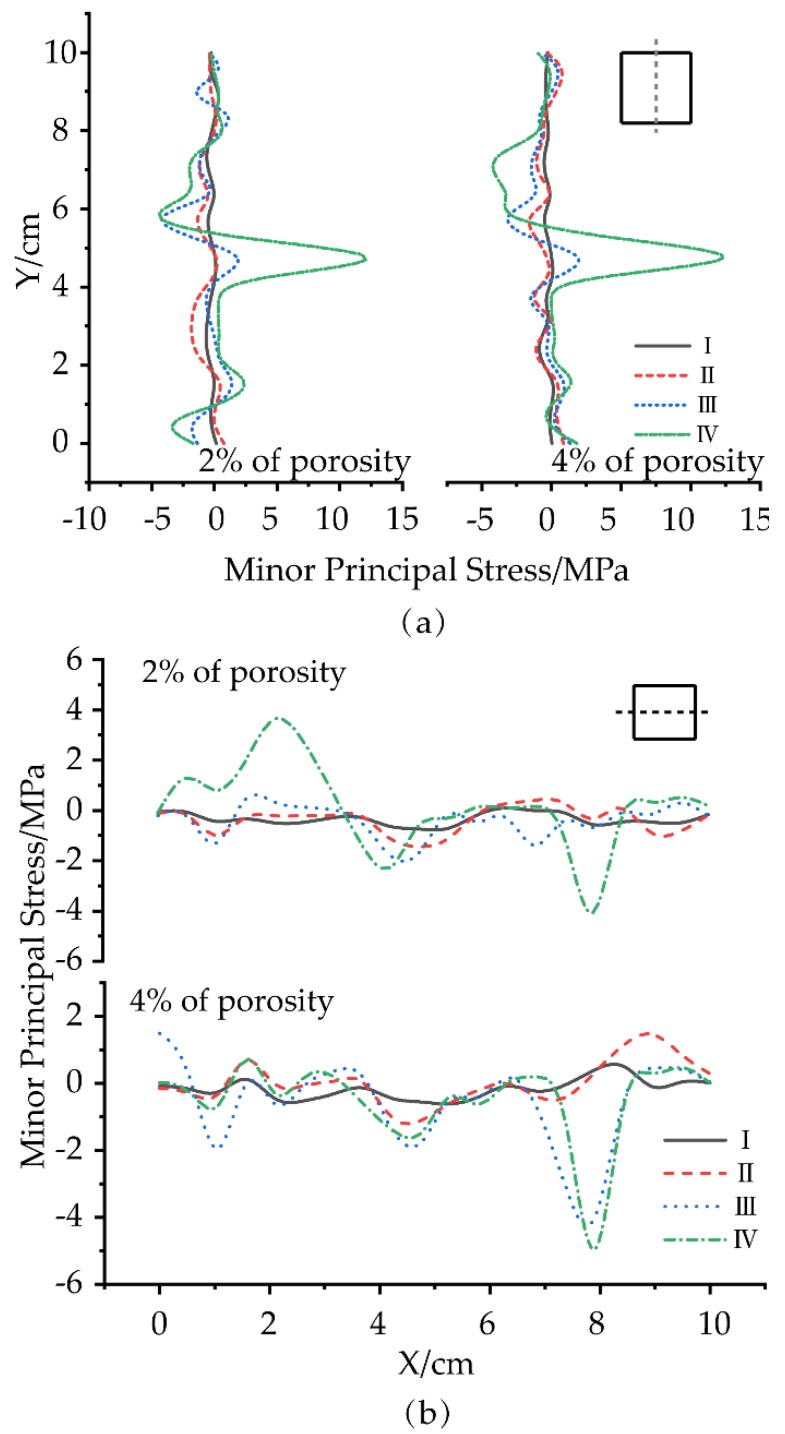

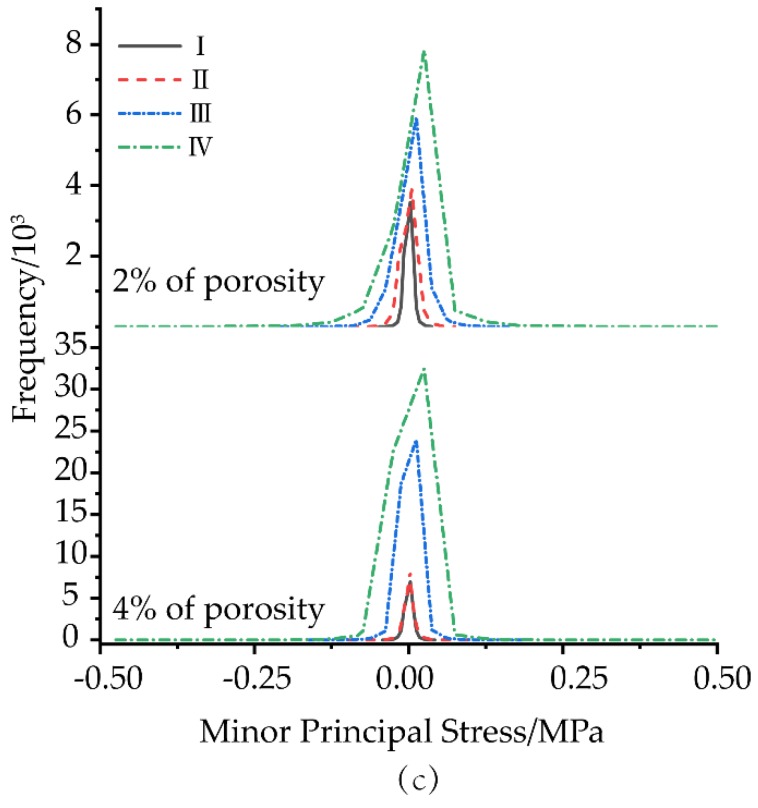
Minor principal stress distribution: (**a**,**b**) in the middle part of models and along the scan line (Where the positive sign is compression and the negative sign is extension) and (**c**) Statistics throughout the model.

Blair and Cook [34,35] indicated that local stress perturbation resulting from the grain-shape heterogeneity might have a significant effect on macroscopic properties. The presence of micropores results in uneven overall stress distribution and rapid stress growth [36,37]. Therefore, models at 2% and 4% porosities were conducted to study the effects of pore and structural anisotropy on the stress distribution at the contact points in various failure stages of concrete, as shown in Figure 11. It was found that the minor principal stress distribution in the middle of the model was detected because it was not significantly affected by the boundary conditions [38].

Especially in Stages I and II, the minor principal stress of the model with a porosity of 2% was more uniform than that of the model with a porosity of 4%. The difference of minor principal stress distribution was more obvious with increasing the uniaxial loading. In each stage, the distribution of minor principal stresses of all contacts was approximately normal. In the case of the same porosity, the dispersion of the minor principal stress became worse as the load proceeded. However, with different porosities, the dispersion of the normal distribution function of the minor principal stress became worse as the load proceeded. In the horizontal direction, more than 70% of the contacts were in the stretched status in the 2% and 4% porosity models. Meanwhile, as the porosity increased, the tensile stress and the proportion increased. Therefore, the increase of the local tensile stress difference caused by geometrical inhomogeneity significantly affected the initiation, growth and nucleation of tensile cracks. In the vertical direction, the difference in the local lateral extension stress caused by the heterogeneity of aggregate and mortar had a great effect on the initiation and propagation of cracks. Furthermore, this effect was the most obvious in Stage IV. The expansion of microcracks gradually affected the distribution of tensile stress as the axial stress increased. Other stresses, such as lateral stresses, can cause the direction of the through the crack to transition toward different compressive stresses, which ultimately affects the damage form of the test specimen.

## 5. Conclusions

This study proposed a method for simulating a mesoscale model using a 2D discrete element program. This method overcame the shortcomings of the traditional GBM method, which is impossible to establish a mesoscopic model with a large difference in grain radius. Moreover, the intrinsic relationships among the grain radius, axial length, direction angle, and elongation were established. The reconstruction of the mesoscale model was achieved based on the maximum degree of similarity of traditional aggregate statistics. By optimizing the aggregate identification process in the CT image, a batch of image processing programs was used to perform probability statistics of each aggregate, including side number, equivalent radius, equivalent radius, and long axis angle. Therefore, a set of methods for aggregate identification, formation and placement is improved.

The initiation and development of micro-cracks in the mesoscale concrete were divided into four processes in uniaxial compression experiments. The results showed that the main type of microcracks was a tensile fracture. Firstly, micro-cracks appeared on the interface between the aggregates of the aggregate. Then, the tensile crack on the contact surface inside the mortar was sharply expanded.

The porosity study indicated that the compressive strength decreased with the increase of porosity. First, the crack appeared at the tip of the aggregate in stage II, and then cracks occurred under two conditions, such as at the aggregate boundary parallel to the direction of the applied load and near the void. Then, the crack was selectively expanded under the axial force. During the loading process, the minor principal stress was normally distributed. As the porosity and loading time increased, the heterogeneity increased.

## Figures and Tables

**Table 1:** First gradation probability distribution parameter in CT slices.

First	Edge Number (*n*)			Elongation Ratio (*R*)			Angle (*µ*)		Equivalent Radius (*r*)	
Type	Gauss			Gauss			mean		mean	
	sigma	mu	R-square	sigma	mu	R-square	min	max	min	max
Variable	2.06	8.24	0.9049	0.97244	1.035	0.99501	−90	90	0.2	0.6

**Table 2:** Second gradation probability distribution parameter in CT slices.

Second	Edge Number (*n*)			Elongation Ratio (*R*)			Angle (*µ*)		Equivalent Radius (*r*)	
Type	Gauss			Log3P1			mean		lognormal	
	sigma	mu	R-square	A	B	C	R-square	min	max	sigma
Variable	1.44	10.37	0.97	0.97	1.03	0.99	0.98	−90	90	0.17

**Table 3:** Third gradation probability distribution parameter in CT slices.

Third	Edge Number (*n*)			Elongation Ratio (*R*)			Angle (*µ*)		Equivalent Radius (*r*)	
Type	Gauss			Log3P1			mean		lognormal	
	sigma	mu	R-square	A	B	C	R-square	min	max	sigma
Variable	0.80	11.87	0.97	50.69	18.726	−1.65	0.98	−90	90	0.35

**Table 4:** Material properties in the numerical model title.

Concrete	Matrix Properties		Contact Properties		Cohesion (MPa)	Dilation Angle (°)	Tensile Strength (MPa)
	Density (kg/m^3^)	*E* (GPa)	*k_n_* (GPa/m)	*k_s_* (GPa/m)			
Mortar	2750 *	38.0 *	24900 ^	9960 ^	3.0	18 *	2.5 *
Aggregate	2880 *	73.0 *	173000 ^	51900 ^	2.8	18 *	6.0 *

Data with “∗” are quoted from Zhou and Liu [28,29], data with “^” are parameters by above formula and the other parameters by repeated trial.
